# Revised Guidelines for the Risk Assessment of Food Additives in Japan

**DOI:** 10.14252/foodsafetyfscj.D-24-00018

**Published:** 2025-03-21

**Authors:** Takahiro Inoue, Daisuke Ikeda, Maako Sugiyama, Asami Suenaga, Minoru Kawashima, Emiko Kondo, Takashi Umemura

**Affiliations:** 1 Food Safety Commission Secretariat, Cabinet Office, Government of Japan, Akasaka Park Bld. 22nd F, Akasaka 5-2-20, Minato-ku, Tokyo 107-6122, Japan; 2 Graduate School of Yamazaki University of Animal Health Technology, 4-7-2 Minami-osawa, Hachiouji,Tokyo 192-0364, Japan

**Keywords:** revised guidelines, risk assessment, food additives, TTC approach, processing aids, breast milk substitutes, Japan

## Abstract

In September 2021, the Food Safety Commission of Japan (FSCJ) revised its 2010 guidelines for the risk assessment of food additives. The revised guidelines, titled Guidelines for the Risk Assessment of Food Additives, incorporate updated international trends in toxicity testing and reflect the experience gained from prior assessments. The revised guidelines are composed of the following 4 chapters: Chapter 1. General Provisions; Chapter 2. Detailed Exposition; Chapter 3. Approach for the risk assessment of processing aids; and Chapter 4. Approach for the risk assessment of additives in foods used as breast milk substitutes for infants under four months old. A central feature of the revised guidelines is the organization of risk assessment into four distinct steps: hazard identification, hazard characterization (toxicological assessment), exposure assessment, and risk characterization. These steps, based on the Codex Alimentarius principles, ensure a thorough and systematic evaluation of food additives. The guidelines also introduce the Threshold of Toxicological Concern (TTC) approach for processing aids and guideline for assessing the additives used as breast milk substitutes for infants under four months old, considering their specific characteristics. This paper provides an overview of these key updates, and offer a structured approach to enhance transparency and consistency risk assessment practices for food additives in Japan.

## 1. Introduction

The food additives*^1^ (hereinafter refer to as “the additives”) which can be used in Japan are classified into designated additives, existing additives, natural flavorings and general food additives. The risk management of the additives has been conducted by the Consumer Affair Agency (CAA) for standards, specifications, guidance and labeling, and the Ministry of Health, Labour and Welfare (MHLW) for monitoring, based on the Food Sanitation Act (Act No. 233 of 1947), while the food safety assessment (the risk assessment) has been implemented by the Food Safety Commission of Japan (FSCJ) since its establishment in 2003 upon request from the risk management agency. The FSCJ has established guidelines for the risk assessment of various foods and related subjects on human health, based on the “Basic Matters” prescribed by Article 21-1 of the Food Safety Basic Act (the Cabinet Decision on June 29, 2012).

Regarding the additives, the FSCJ has created “Guideline for Assessment of the Effect of Food on Human Health Regarding Food Additives” on May 27, 2010 (hereinafter refer to as “Guideline 2010”) based on the former experience of the risk assessments in Japan and concept of domestic and international safety evaluation, and the FSCJ decided that the “Guideline 2010” should be followed whenever risk assessment of additives had been conducted. Afterwards, the FSCJ has established “Guidelines for the Assessment of Flavoring Substances in Foods on Health” (Decision of the Committee on May 17, 2016), “Guidelines for the Risk Assessment of Additives (Enzymes) in Foods” (Decision of the Committee on July 18, 2017), and “Guidelines for the Risk Assessment of Food Additives for Fortification” (Decision of the Committee on July 18, 2017), for the safety assessment of flavoring substances, enzyme additives, and additives for fortifications, respectively. Consequently, the FSCJ decided to apply the “Guideline 2010” to the risk assessments of additives other than flavoring substances, enzymes and additives for fortification after establishment of those guidelines. In addition, the assessment concept for processing-aids used as sterilizers and extraction solvents was compiled and added as an appendix: “Stance for the Risk Assessment of Processing Aids (Food Disinfectants and Extractants)” to the “Guideline 2010” (Decision of the Committee on July 18, 2017).

In September 2021, approximately ten years after the “Guideline 2010” was established, a comprehensive revision of the guidelines was undertaken^1^^) *2^. The revised guidelines (hereafter refer to as “Revised Guidelines for Additives”) incorporated the latest international trends in risk assessment, toxicity testing, as well as the knowledge gained from the previous risk assessments of the additives based on the “Guideline 2010”. In this revision, two new chapters, Chapter 3 and Chapter 4, were added. Chapter 3 introduced the Threshold of Toxicological Concern (TTC) approach to the risk assessment of processing aids. The TTC approach has been internationally adopted as a method for assessing low-dose exposure to chemicals and has already been introduced by the Food Safety Commission of Japan (FSCJ) for the risk assessment of flavoring agents. Chapter 4 addressed the assessment of the additives used as breast milk substitutes for infants under four months old. This evaluation takes into account the fact that infants appear to have different characteristics from adults in terms of absorption, distribution, metabolism, and excretion mechanisms of external chemical substances, as well as differences in sensitivity to these substances. 

This paper provides an overview of the “Revised Guidelines for Additives”, focusing on the main points of revision and their background. Chapters 3 and 4, which were newly added, are described in detail in Sections 2.6 and 2.7, respectively. Additionally, future challenges and other related issues are also discussed.

## 2. Overview of the “Revised Guidelines for Additives”

### 2.1. Structure of the “Revised Guidelines for Additives”

The “Revised Guidelines for Additives” are composed of the following 4 chapters: Chapter 1. General Provisions; Chapter 2. Detailed Exposition; Chapter 3. Approach for the risk assessment of processing aids; and Chapter 4. Approach for the risk assessment of the additives used as breast milk substitutes for infants under four months old. After these 4 chapters, an appendix: a table of the documents needed for the risk assessment, and the following references: (1) a glossary of terminology^2^^)^; and (2) related documents^3^^,^^4^^,^^5^^,^^6^^)^, are attached (**Table 1**).

**Table 1.** Structure of "Revised Guidelines for Additives"

Chapter 1. General Provisions	
Article 1. Background Article 2. Purpose Article 3. Definition Article 4. Approach for the Risk Assessment of Food Additives Article 5. Approach for the Documents required for the Risk Assessment	Article 6. The Risk Assessment Article 7. Revision of the Risk Assessment Article 8. Revision of this Guideline Article 9. The Risk Assessment of Flavoring Substances Article 10. The Risk Assessment of Enzymes Article 11. The Risk Assessment of Additives for Fortification
Chapter 2. Detailed Exposition	
Article 1. Outline of the additives to be assessed Article 2. Findings regarding the Safety Article 3. Estimation and consideration of the Daily Intake	
Chapter 3. Approach for the Risk Assessment of Processing Aids	
Article 1. Scope of application Article 2. Risk assessment procedure Article 3. Classification criteria for the Estimated Daily Intake	Article 4. Toxicological assessment Article 5. Risk Characterization Article 6. Substances and toxic effects requiring special consideration
Chapter 4. Approach for the risk assessment of additives in foods used as breast milk substitutes for infants under four months old	
Article 1. Scope of application Article 2. Toxicological assessment Article 3. Exposure Assessment Article 4. Risk Characterization	
Appendix: Documents that are required to the risk assessment of additives for designation request Reference Article 1. A glossary of terminology^2^^)^ Article 2. Related documents^3^^,^^4^^,^^5^^,^^6^^)^	

### 2.2. Purpose

The “Revised Guidelines for Additives” aim to establish the guiding principles of risk assessment on the additives and define the scope of required data, when designating additives that do not pose a risk to human health or when revising standards for additives, as stipulated by the Food Sanitation Act.

### 2.3. Scope

The “Revised Guidelines for Additives” apply to food additives other than flavoring substances^7^^)^, enzymes^8^^)^, and the additives for fortification^9^^)^, which have their separate guidelines.

### 2.4. Basic Steps of Food Risk Assessment in the “Revised Guidelines for Additives”

**Fig. 1.**
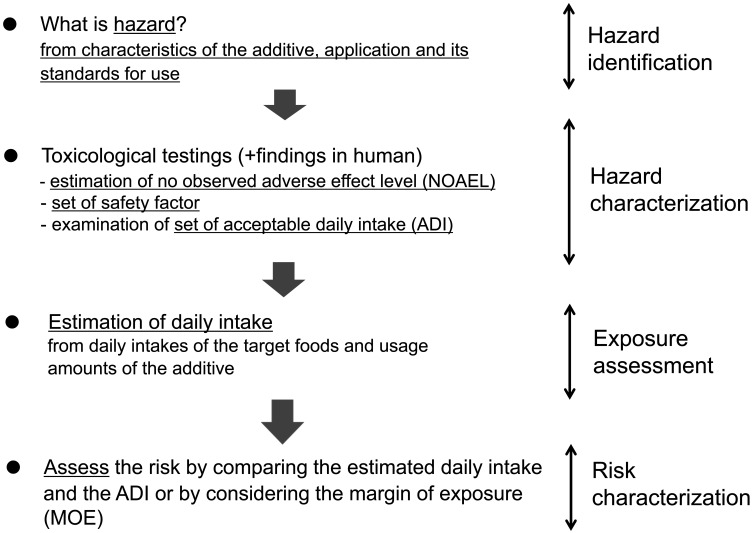
Steps of Risk Assessment of the Additives In actual assessment, the order of hazard characterization and exposure assessment may vary.

The “Revised Guidelines for Additives” organize the basic steps for food risk assessment of the additives based on the four stages outlined in the “Working Principles for Risk Analysis for Food Safety for Application by Governments (CAC/GL 62-2007)”: 1) Hazard identification; 2) Hazard characterization; 3) Exposure assessment; and 4) Risk characterization (**Fig. 1**).

Although “Hazard identification” is not a term used in the “Revised Guidelines for Additives”, it refers to the stage of identifying substances that may pose toxic effects on human health based on the characteristics of the additive and its standards for use. In the case of additives, this stage is particularly important if the additive undergoes chemical changes in the food to which it is added.

“Hazard characterization” is referred to as “Toxicological assessment” in the “Revised Guidelines for Additives”. This is usually based on data from toxicokinetics (absorption, distribution, metabolism, and excretion) and toxicity tests (genotoxicity, repeated dose toxicity, carcinogenicity, reproductive toxicity, developmental toxicity, allergenicity, and other tests) using animals. However, in recent years, if human data is available, it is given more weight in the safety assessment.

The “Guideline 2010” referred to “Exposure assessment” as “Estimation of daily intake”, and its description included content that should be interpreted as part of “Risk characterization”. The “Revised Guidelines for Additives” clarify that this step is strictly the “Estimation of daily intake”. In addition, it should be noted that in actual risk assessment, the “Estimation of daily intake” step may be carried out in parallel with the “Toxicological assessment” step.

The guidelines state that the daily intake should be estimated taking care to avoid without underestimate, and that in principle, the daily intake of an additive is estimated by multiplying the daily intake of the target food by the additive usage amount. If the ingredients contained in the additive are also ingredients that intake from a normal diet (for example, calcium ions), the risk assessment should also take into account the estimated intake from a normal diet. Furthermore, considering the need to assess cases where intake varies significantly among populations, the “Revised Guidelines for Additives” add the following statement that the estimated daily intake in the relevant specific population shall be considered if more appropriate estimation is possible.

“Risk characterization” is the step in which the results of the risk assessment are judged based on the preceding three steps. The “Revised Guidelines for Additives” categorize the outcomes into the following four cases.

(I) “The Acceptable Daily Intake (ADI) is specified to be XX.”
The phrase is used when specification of the ADI is considered to be appropriate as the results of the risk characterization based on the characteristics of the additive, standards for use, toxicological assessment, exposure assessment and so on.

(II) “It is unnecessary to specify the ADI, since the assessed item is considered to have no safety concern as long as it is used appropriately as an additive.”
The phrase is for the case where the ADI is not specified despite that it can be specified, in accordance with the conclusion that the toxicity of the assessed additive is considerably low or the Estimated daily intake is sufficiently low compared to the NOAEL.

(III) “The assessed item is of no concern for food safety as long as used appropriately as a food additive.”
The phrase is for the case where the Margin of Exposure (MOE) is assessed for the additive that does not fit with the concept of ADI.

(IV) “The ADI cannot be specified.”
The phrase is for the case where the ADI cannot be specified for the additive or other substance that is evaluated as a genotoxic carcinogen.

### 2.5. Main Revision Points in the Step of Toxicological Assessment

(1) Clarification of toxicity test methods
Toxicity tests required for the assessment are described including the specific provisions in the Article 2 of the Chapter 2. Examples such as OECD Test Guidelines are added to clarify these requirements further in the “Revised Guidelines for Additives”.

(2) Animal species in repeated dose toxicity tests
The “Guideline 2010” required testing on non-rodent species. In the “Revised Guidelines for Additives”, it is not strictly necessary to use non-rodent species, allowing for flexibility in the selection of species, such as one rodent species (typically rats) and one non-rodent species (typically dogs), or two rodent species (typically rats and mice).

(3) Allergenicity tests
For allergenicity testing, it was prescribed in this amendment that alternative methods which is alternative to conventional study methods using animals shall be useful if assessment is conducted with defined approach by Integrated Approaches to Testing and Assessment (IATA) based on Adverse Outcome Pathway (AOP), showing examples of such alternative methods. Regarding allergenicity tests, it has been prescribed that the potential allergenicity shall be examined with adequate sensitization and induction methods if allergenicity is suspected in consideration of the findings on the additives and the usage. In other words, allergenicity test is not necessarily required for every additive thus the potential allergenicity needs to be examined at first.

(4) General pharmacological tests
In consideration of the experience of previous risk assessments of the additives and pesticides registration, the “Revised Guidelines for Additives” amend the requirement for general pharmacological test from being mandatory to being required only when necessary.

### 2.6 Approach for the Risk Assessment of Processing Aids (the Chapter 3 in the Revised Guidelines)

Processing aids designates the substance that meets one of the following conditions, among food additives used in food processing.

1) An additive that is removed from the food before final packaging.

2) An additive that is converted to a naturally contained component in food, and the amount of the component does not significantly increase the amount of the natural component.

The “Guideline 2010” included an annex specifying sterilizing agents and extraction solvents used as processing aids. However, the “Revised Guidelines for Additives” introduce their Chapter 3, which expands the scope to all processing aids including their impurities, side products and degradants, for which an estimated daily intake could be calculated. Furthermore, recognizing that processing aids are distinct from other additives because they generally do not remain in the final food product and have a minimal exposure level, the revised guidelines adopt the TTC approach. This approach sets the required testing items progressively according to the estimated intake levels.

#### 2.6.1 Classification of estimated intake levels

The classification of estimated intake levels for the substance under evaluation is, in principle, determined by matching the estimated daily intake with the estimated intake range (**Table 2**). However, if the estimation method results in an excessively high estimate of daily intake, a comprehensive judgment may be made. For instance, if an excessively high estimate places the intake near the boundary of the estimated intake range, the classification should not be determined mechanically but instead be assessed based on individual characteristics and other relevant factors.

**Table 2.** Classification of Estimated Intake Range

Estimated Intake Range	
Class a	90 μg/person/day or less
Class b	More than 90 μg/person/day 2,000 μg/person/day or less
Class c	More than 2,000 μg/person/day

#### 2.6.2 Estimated intake range of each class

Umemura et al*.*^6^^)^ reported that it is appropriate to set the concentration ranges for each category of processing aids based on the standards of the “Guidelines for Risk Assessment of Food Apparatus, Containers and Packaging”^10^^)^. The boundary value between [class a] and [class b] in the “ Guidelines for Food Apparatus, Containers and Packaging” is a concentration of 0.05 mg/kg in food; however, the value results from rounding 0.045 mg/kg to one significant figure. When the pre-rounded value (i.e., 0.045 mg/kg) is used to calculate the daily intake per person, it yields the result shown in Equation 1, and the chapter 3 adopts the value of 90 µg/person/day obtained from Equation 1. This value is the same as the TTC value for Class III of Cramer classification.

Equation 1. Daily Intake per person: 0.045 mg/kg × 2 kg
(food) = 90 μg/person/day

The boundary value between [class b] and [class c] for the “Guidelines for the Risk Assessment of Food Apparatus, Containers and Packing” is 1.0 mg/kg. This value was established considering international harmonization and was not rounded to effective digits. Therefore, the daily intake per person is calculated by Equation 2 using 1.0 mg/kg as it is. Thus 2,000 μg/person/day is obtained, and the value was employed in the “Revised Guidelines for Additives”.

Equation 2. Daily Intake per person:
1.0 mg/kg × 2 kg (food) = 2,000 μg/person/day

#### 2.6.3 Hazard characterization and risk characterization

In principle, the toxicity of the substance under evaluation should be assessed based on the results of various toxicity tests required for each estimated intake level (**Table 3**). However, if a substance or toxic effect is identified in the Article 6 of the Chapter 3 as requiring special attention*^3^, additional test results listed in that section shall also be considered.

**Table 3.** Testing required by each class of Estimated Intake Range

Estimated Intake Range		Testing
Class a	90 μg/person/day or less	Genotoxicity test
Class b	More than 90 μg/person/day 2,000 μg/person/day or less	Genotoxicity test Subacute (Subchronic*) toxicity test *90-day repeated dose toxicity study is applicable.
Class c	More than 2,000 μg/person/day	Toxicokinetics test Genotoxicity test Repeated dose toxicity test Carcinogenisity test Reproductive toxicity test Developmental toxicity test Allergenicity test

Note: In addition to the testing items in Table 2, available information of the target substance regardless of class of Estimated Intake Range (particularly results of toxicity tests other than the tests required for each class) is demanded to be collected and provided.

If the estimated intake level of the substance under evaluation is “Class-a”, risk assessment should be conducted based on the results of genotoxicity tests. If the estimated intake level is “Class b” or “Class c”, the estimated daily intake of the substance should be compared with the NOAEL or other relevant values for the substance in toxicity tests, in order to estimate the level of risk to human health for the population exposed to the substance.

### 2.7 Approach for the Risk Assessment of Additives in Foods Used as Breast Milk Substitutes for Infants under Four Months Old (the Chapter 4 in the Revised Guidelines)

For infants under four months old, breast milk or infant formula is the sole source of nutrition. The absorption, distribution, metabolism, excretion mechanisms and sensitivity to external chemical substances in infants are believed to differ from those in adults. Therefore, it is necessary to conduct the risk assessment for the additives used as breast milk substitutes infants under four months old, taking into account their specific characteristics. Accordingly, the “Revised Guidelines for Additives” summarize the evaluation approach for these additives in their Chapter 4.

#### 2.7.1 Scope of the Chapter 4

For the additives ingested by infants whose sole source of nutrition is breast milk or infant formula, it is important to consider the potential for relatively high doses to be ingested within a short period. JECFA specifies the infant age up to 12 weeks^11^^,^^12^^)^, and EFSA sets it up to 16 weeks^13^^)^. However, the scope of this chapter defines the applicable age as “under four months old” without uniformly setting the age in weeks. Meanwhile, even if the food is a breast milk substitute and infants over five months of age consume it, if infants under four months old can also consume it, the provisions of this chapter apply to the additives used in such foods. However, the “Revised Guidelines for Additives” do not apply to the additives for fortification such as vitamins and minerals; thus, if an additive is related to nutritional components, this chapter does not apply.

#### 2.7.2 Toxicological assessment

Chapter 4 of the revised guidelines states that, where appropriate, toxicokinetic studies and toxicity tests on young animals (minipigs) should also be used in the evaluation. Appropriate test data (clinical research, post-marketing surveillance, etc.) on the additives and related substances being evaluated should be used where available.

#### 2.7.3 Exposure assessment

Regarding exposure assessment, the intake of the additives from the target food is, in principle, calculated by multiplying the maximum allowable amount of the additive in the target food by the daily intake of the target food. In this chapter, a uniform method for estimating daily intake is not specified. However, the report by the Japanese Dietary Reference Intakes (DRIs) Working Group notes that artificially fed infants generally have a higher total energy expenditure than breastfed infants^14^^)^. Therefore, careful consideration is necessary when deciding whether to directly use the standard feeding volume presented in the report to estimate daily intake.

#### 2.7.4 Risk characterization

The “Revised Guidelines for Additives” state that for additives for which “it is not necessary to specify an ADI.”, an MOE (Margin of Exposure) assessment should, in principle, be conducted based on the results of juvenile animal studies. For the additives with an established ADI or similar considerations, individual assessments should be conducted.

## 3. Discussion

In “Revised Guidelines for Additives”, the steps of risk assessment, which were not unified in “Guideline 2010”, have been reorganized into four stages. We expect that this reorganization standardizes both the procedure of risk assessment and the risk assessment reports. Additionally, in “Revised Guidelines for Additives”, the expression of results risk characterization was categorized into four classifications. Based on these classification, 84 cases of the additives (excluding flavoring substances) risk assessments conducted by the Food Safety Commission between July 2003 and August 2024 (counting repeated evaluations of the same substance as a single case) were categorized as follows: 40 cases fell into Category I “The Acceptable Daily Intake (ADI) is specified to be XX.”, while 36 and 7 cases were classified under Category II “It is unnecessary to specify the ADI, since the assessed item is considered to have no safety concern as long as it is used appropriately as an additive.” and Category III “The assessed item is of no concern for food safety as long as used appropriately as a food additive.”, respectively. And only one case (Madder color*^4^^)^ corresponded to the Category IV “The ADI cannot be specified.”. These are indicated that the ADI may not need to be specified for some substances used as the additives, in other words, no numerical ADI is considered necessary. This may be the case when the additive is determined to be of very low toxicity, based on the biological and toxicological data, and the total dietary intake of the additives, arising from the levels used in foods to achieve the desired function, does not represent a hazard^15^^)^.

In “Revised Guidelines for Additives”, allergenicity testing was made more flexible, allowing the use of non-animal testing methods if they follow the defined approaches by IATA. Regarding processing aids, the TTC approach was incorporated. The EFSA guidance for the TTC approach in food safety assessment states that the TTC approach is a pragmatic, scientifically valid methodology to assess the safety of substances of unknown toxicity found in food^16^^)^. This introduced the tiered approach to determine the required toxicological testing items, based on the estimated daily intake levels, as a useful screening tool. However, as of August 2024, no test data based on reduced toxicological testing requirements have been submitted. Similarly, there have been no submissions of juvenile animal testing data for the additives used in breast milk substitutes intended primarily for infants up to approximately four months of age. Nonetheless, it is anticipated that future applications will be submitted in accordance with the revised guidelines and the corresponding data sets.

## 4. Conclusion

In “Revised Guidelines for Additives”, Chapter 3 was prepared with reference to the approaches of JECFA and EFSA in line with international trends in the risk assessment, where the new concept of the TTC approach has had a big impact, particularly for the risk assessment of the exposure to a trace amount.

And there are no cases where Chapter 4 of these guidelines has been applied to the evaluation of the additives used in breast-milk substitutes, but in the future, when evaluating additives used in infant formula, etc., more appropriate evaluations should be carried out, taking into account the physiological differences between adults and infants.

The FSCJ is conducting support programs for researchers aimed at updating risk assessments for the additives in the future. These programs include research on the development of alternatives to animal testing, improvements in exposure assessment for the additives, development of risk assessment methods for food safety in high-risk populations, and examinations of risk assessment methods for nanomaterials. Going forward, the aim is to utilize the outcomes of these research initiatives to implement further revisions to the guidelines.

## Note

^*1^ The substances listed by the Prime Minister as having been generally served for human consumption and used as the additives based on the Article 12 of the Food Sanitation Act.

^*2^ “Revised Guidelines for Additives’’ had been already revised again in April 2024. This revision was due to a change in the ministry responsible for some parts of the Food Sanitation Law.

^*3^ In the risk assessment of additives, substances that require special consideration include highly reactive substances such as disinfectant, metals, inorganics, and proteins. In addition, toxic effects that require special consideration include neurotoxicity, immunotoxicity and endocrine activity.

^*4^ https://www.fsc.go.jp/hyouka/hy/hy-maddercolor-hyouka-translation.pdf
